# PVA/Gelatin/*Cnidium monnieri* Composite Scaffolds for Atopic Dermatitis Skin Tissue Regeneration

**DOI:** 10.3390/gels11020143

**Published:** 2025-02-18

**Authors:** Young Ho Seo, Sun Young Park, Sangmin Lee, Myunghoo Kim, Seon Beom Kim, Tae Hwan Oh

**Affiliations:** 1 Institute for Future Earth, Pusan National University, Busan 46241, Republic of Korea; seoyh@pusan.ac.kr (Y.H.S.); psy731@pusan.ac.kr (S.Y.P.); fapnu.sml@gmail.com (S.L.); mhkim18@pusan.ac.kr (M.K.); sbkim@pusan.ac.kr (S.B.K.); 2 Department of Food Science & Technology, Pusan National University, Miryang 50463, Republic of Korea; 3 Department of Animal Science, Pusan National University, Miryang 50463, Republic of Korea; 4 School of Chemical Engineering, Yeungnam University, Gyeongsan 38541, Republic of Korea

**Keywords:** atopic dermatitis, polyvinyl alcohol, gelatin, *Cnidium monnieri*, scaffold, inflammation

## Abstract

Atopic dermatitis (AD) is a chronic inflammatory skin condition characterized by impaired barrier function and persistent inflammation, necessitating advanced therapeutic solutions. This study presents the development of a novel composite hydrogel scaffold composed of polyvinyl alcohol (PVA), gelatin, and *Cnidium monnieri* (CM) extract, designed to address the dual challenges of tissue regeneration and inflammation suppression. Fabricated via optimized freeze–thaw crosslinking and lyophilization, the scaffold exhibited a highly porous structure conducive to enhanced cell proliferation and controlled bioactive release. FT-IR analysis confirmed robust intermolecular interactions among PVA, gelatin, and CM bioactives, while SEM imaging revealed a well-developed porous network. The UPLC analysis demonstrated the sustained release of key CM compounds, such as osthole and imperatorin, which contributed to the scaffold’s anti-inflammatory properties. Biological assessments using HaCaT keratinocytes under inflammatory conditions induced by TNF-α and IFN-γ revealed improved cell viability and significant suppression of IL-8 expression, a critical marker in AD-related inflammation. These findings underscore the potential of the PVA/Gel/CM composite hydrogel as an advanced therapeutic platform for inflammatory skin disorders.

## 1. Introduction

Atopic dermatitis (AD) is a chronic, inflammatory skin disorder with a global prevalence of up to 20% in children and 10% in adults. Its complex etiology involves genetic predisposition, environmental factors, and immune dysregulation, leading to disrupted skin barrier function, persistent inflammation, and pruritus [1,2,3]. The impaired barrier increases trans-epidermal water loss and susceptibility to allergens and pathogens, exacerbating disease severity and the risk of secondary infections [4,5]. Current treatments, including corticosteroids and calcineurin inhibitors, often fail to provide long-term relief due to side effects and frequent relapses [6]. This highlights the pressing need for innovative therapeutic approaches that address both inflammation and barrier repair [7,8].

Tissue engineering offers a promising solution by developing biomimetic scaffolds to restore the extracellular matrix (ECM), support cell adhesion, and deliver bioactive compounds [9]. Polyvinyl alcohol (PVA), a widely used synthetic polymer in biomedical applications, is favored for its biocompatibility, chemical stability, and mechanical tunability [10,11]. However, its limited protein adsorption and cell adhesion properties necessitate functionalization with natural polymers [12]. Gelatin, a collagen derivative, complements PVA by mimicking ECM components, enhancing cell adhesion, and promoting proliferation [13]. Its bioactive Arg-Gly-Asp (RGD) sequences further support integrin-mediated cell attachment and tissue regeneration [14]. To further enhance the mechanical strength, biocompatibility, and bioactivity of scaffolds, various natural polymers, such as alginate, chitosan, hyaluronic acid, and silk fibroin, have been explored alongside gelatin for tissue regeneration applications [15].

Incorporating bioactive natural compounds into scaffolds has emerged as a promising strategy to enhance therapeutic efficacy [16,17]. *Cnidium monnieri* (CM), a traditional East Asian medicinal herb, is known for its potent anti-inflammatory, antioxidant, and antimicrobial properties. Its bioactive components, including osthole, imperatorin, and xanthotoxin, regulate inflammatory signaling and promote tissue repair [18,19,20,21]. Despite its therapeutic potential, CM’s integration into tissue engineering remains underexplored.

This study introduces a novel PVA/gelatin/CM composite hydrogel scaffold fabricated via repeated freeze–thaw cycles and lyophilization, designed to address the dual challenges of tissue regeneration and inflammation suppression in AD. The scaffold’s porous structure supports cell proliferation while enabling the sustained release of CM bioactives with anti-inflammatory properties. Interleukin-8 (IL-8), a key chemokine that amplifies inflammatory responses by recruiting and activating neutrophils, plays a pivotal role in the pathogenesis of AD [22], with elevated levels in keratinocytes, such as HaCaT cells, exacerbating inflammation in response to cytokines, such as TNF-α and IFN-γ, making its reduction a critical target for mitigating AD-related inflammation.

Comprehensive characterization using FT-IR, SEM, and UPLC analysis, along with biological assessments on HaCaT human keratinocytes, demonstrated its biocompatibility and therapeutic potential, including its ability to reduce IL-8 levels. By addressing critical gaps in AD management, this study provides a versatile platform for integrating bioactive compounds into regenerative scaffolds, with potential applications for broader inflammatory conditions.

## 2. Results and Discussion

### 2.1. FT-IR and Mechanical Properties

**Figure 1:**
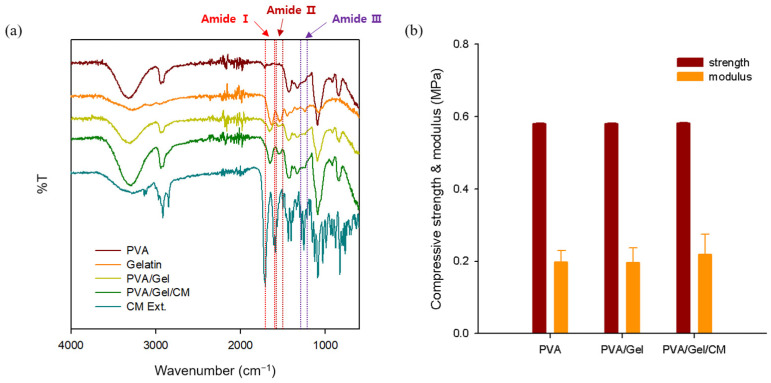
FTIR spectra (**a**); and compressive strength and modulus analysis (**b**), of PVA/Gel and PVA/Gel/CM hydrogel scaffolds.

Figure 1a presents the FT-IR spectra of PVA/Gel and PVA/Gel/CM hydrogels, highlighting the chemical interactions between PVA, gelatin, and CM. In the range of 3200–3500 cm^−1^, broad absorption peaks corresponding to O–H and N–H stretching vibrations were observed, indicative of hydroxyl groups from PVA and amine groups from gelatin. The incorporation of CM resulted in a noticeable broadening and increased intensity of these peaks, suggesting enhanced hydrogen-bonding interactions among PVA, gelatin, and CM bioactive compounds. This phenomenon can be attributed to the presence of phenolic hydroxyl groups in CM, which further reinforce the hydrogen-bonding network within the hydrogel matrix.

In the region of 1600–1700 cm^−1^, associated with the C=O stretching vibrations (Amide I) of gelatin, slight shifts were observed in the PVA/Gel/CM hydrogel, indicating modifications in the chemical environment due to interactions between CM bioactive compounds (e.g., osthole and imperatorin) and the polymer matrix. The presence of phenolic compounds and aromatic ring structures in CM also contributed to these spectral shifts, reinforcing the hydrogel’s internal interactions. Similarly, spectral variations were observed in the 1500–1600 cm^−1^ range, corresponding to N–H bending and C–N stretching vibrations (Amide II), which exhibited minor shifts in intensity and peak position. These changes further support the hypothesis of molecular interactions between CM bioactive compounds and the amide bonds in gelatin. Additionally, in the 1200–1300 cm^−1^ region, associated with C–N and N–H bending vibrations (Amide III), subtle changes in peak intensity were noted, reflecting the influence of CM on the bonding environment within the hydrogel matrix.

These spectral modifications collectively confirm that the incorporation of CM bioactive compounds, including phenolic and aromatic structures, significantly alters the chemical interactions and structural organization of the PVA/Gel composite. The observed molecular interactions contribute to enhanced hydrogen bonding, modifications in the amide environment, and the introduction of aromatic contributions, which ultimately improve the gelation properties and structural stability of the hydrogel. Overall, the FT-IR analysis reveals critical structural changes driven by hydrogen-bonding and chemical interactions, which enhance the gelation characteristics and stability of the PVA/Gel/CM scaffold. These modifications are particularly relevant for biomedical applications, where controlled bioactive release and robust mechanical properties are required.

Figure 1b presents the compressive strength and modulus of the PVA, PVA/Gel, and PVA/Gel/CM hydrogel scaffolds. The measured compressive strength for all compositions was approximately 0.58 MPa. In terms of compressive modulus, the PVA/Gel/CM scaffold exhibited a slight increase compared to the PVA and PVA/Gel, suggesting potential interactions between CM components and the polymer network that may contribute to a more cohesive matrix. This indicates that the addition of gelatin and CM to the PVA matrix does not significantly affect the mechanical strength, while the slight increase in modulus demonstrates that despite the incorporation of CM bioactive compounds, the structural integrity and mechanical stability of the scaffold are maintained.

### 2.2. Result of Gel Content and Swelling Ratio

**Figure 2:**
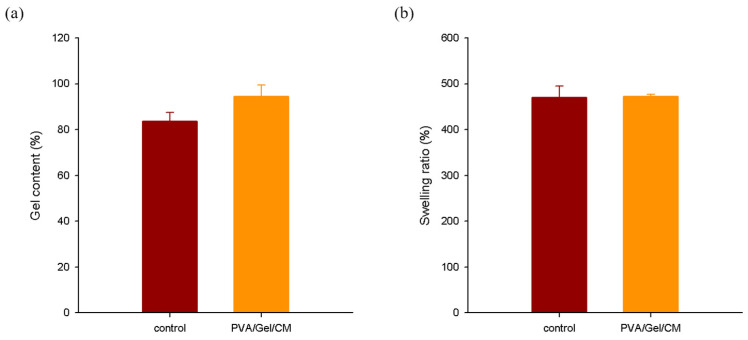
Gel content (**a**); and swelling ratio (**b**), of PVA/Gel (control) and PVA/Gel/CM hydrogels.

Figure 2 illustrates the gelation rate and swelling ratio of the PVA/Gel/CM composite hydrogel. The results show that the hydrogel containing the CM extract achieved a 12.8% higher gelation rate compared to the control group, attributed to the bioactive compounds present in CM. These compounds, particularly osthole and imperatorin, likely enhance intermolecular interactions between PVA and gelatin, resulting in a more robust three-dimensional gel network. The functional groups of these bioactives, such as hydroxyl (–OH) and carbonyl (–C=O) groups, form additional hydrogen bonds with the hydroxyl groups in PVA and gelatin, contributing to increased gelation efficiency.

In contrast, the swelling ratio of the composite hydrogel showed no significant difference compared to the control group, indicating that the inclusion of CM does not adversely affect the hydrogel’s swelling behavior. This consistency suggests that the structural modifications introduced by CM maintain the scaffold’s ability to absorb water, a critical factor for cell proliferation and bioactive compound delivery.

These findings emphasize the dual benefits of incorporating CM into the hydrogel matrix: enhanced gelation efficiency and preserved swelling capacity. This optimized combination ensures the hydrogel’s suitability for tissue engineering applications by balancing structural integrity with functional adaptability [22,23,24].

### 2.3. SEM

**Figure 3:**
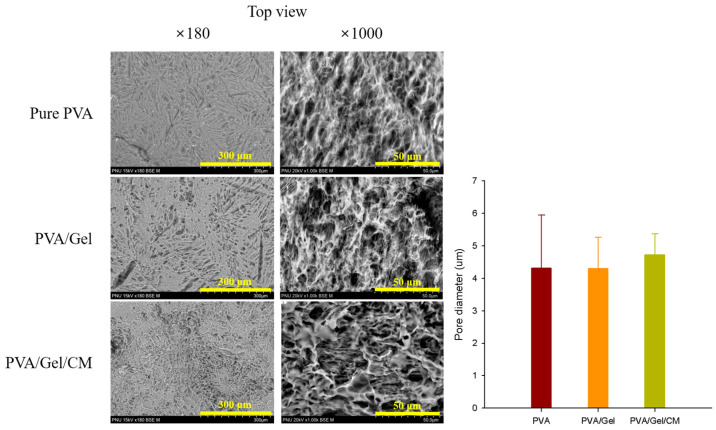
SEM image and pore size of the pure PVA, PVA/Gel, and PVA/Gel/CM hydrogel scaffolds. The scale bars represent 300 μm (×180) and 50 μm (×1000).

Figure 3 shows the SEM images of the cross-sectional and lateral views of the pure PVA, PVA/Gel, and PVA/Gel/CM hydrogels, revealing distinct differences in surface morphology and porous structure among the samples.

The pure PVA hydrogel exhibited a relatively smooth and homogeneous surface with limited porosity, indicative of a less developed three-dimensional network. The addition of gelatin significantly altered the surface morphology, resulting in a rougher and more irregular texture, as well as enhanced porosity. This transformation is attributed to the intrinsic properties of gelatin, which promote the formation of a three-dimensional porous network due to its interactions with PVA during freeze–thaw crosslinking.

The PVA/Gel/CM composite hydrogel demonstrated the most well-developed and complex porous structure, characterized by interconnected pores with enhanced depth and uniformity. This improvement is likely due to the incorporation of CM bioactive compounds, such as osthole and imperatorin, which may facilitate molecular interactions that promote a denser and more stable network. The enhanced porosity observed in the PVA/Gel/CM hydrogel supports its ability to retain and release bioactive compounds efficiently, while also providing an optimal environment for cell proliferation and nutrient exchange.

The highly porous architecture of the PVA/Gel/CM hydrogel is critical for its intended biomedical applications, as it facilitates swelling, bioactive compound delivery, and cellular infiltration. The structural modifications introduced by gelatin and CM collectively enhance the scaffold’s functionality, making it a promising candidate for tissue engineering applications, particularly in the treatment of inflammatory skin disorders like atopic dermatitis.

### 2.4. UPLC Analysis and Release Profile

**Figure 4:**
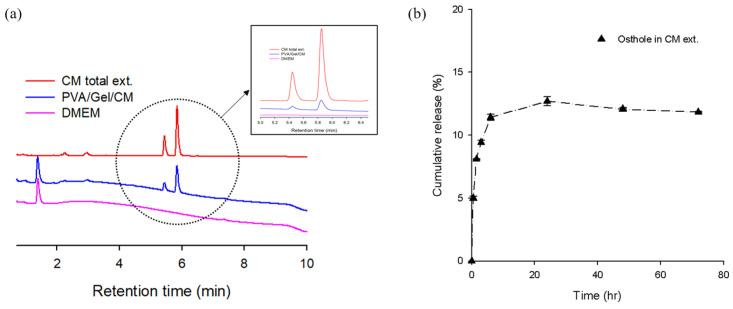
UPLC chromatogram (**a**) of CM total extract, DMEM medium, and DMEM medium after 1 day of incubation with the PVA/Gel/CM scaffold; and cumulative release rate (**b**) of PVA/Gel/CM scaffold.

Figure 4 presents the UPLC analysis results for the following three samples: the total extract of CM; the pure DMEM medium; and the DMEM medium incubated with the PVA/Gel/CM scaffold for 24 h. This experiment aimed to evaluate the release profile of bioactive compounds, such as osthole and imperatorin, from the PVA/Gel/CM scaffold into the medium.

The UPLC chromatogram of the total CM extract exhibited two prominent peaks at retention times (R.T.) of 5.4 min and 5.8 min, corresponding to imperatorin and osthole, respectively. These characteristic peaks were also detected in the DMEM medium after incubation with the PVA/Gel/CM scaffold but were absent in the pure DMEM medium, confirming the successful release of bioactive compounds from the scaffold into the medium. The controlled release behavior observed in the scaffold is attributed to its porous architecture, which facilitates the gradual diffusion of CM components. The combination of optimized freeze–thaw crosslinking and lyophilization techniques likely enhanced the scaffold’s capacity to retain and subsequently release bioactive compounds, ensuring a sustained therapeutic effect.

The release kinetics study revealed that CM exhibited a rapid-release phase within the first 12 h, primarily due to the swelling behavior of the highly porous scaffold and the concentration-driven diffusion mechanism. This initial burst release is likely a result of the lyophilization-induced porous structure, which enables immediate hydration and subsequent diffusion of the bioactive compounds. Beyond 24 h, the release rate plateaued, indicating a stable and sustained release profile. The UPLC analysis confirmed that osthole, the major bioactive component in CM, was used as the reference compound; at a retention time of 5.8 min, the final cumulative release rate of osthole in the CM extract was determined to be approximately 12.7%.

These findings highlight the potential of the PVA/Gel/CM scaffold as an effective delivery system for bioactive compounds, particularly for applications requiring localized and controlled release. Furthermore, the sustained release of osthole and imperatorin suggests that the scaffold may be well-suited for therapeutic applications in inflammatory conditions, such as atopic dermatitis, where prolonged exposure to anti-inflammatory agents is crucial for optimal therapeutic efficacy.

### 2.5. Cell Cytotoxicity

**Figure 5:**
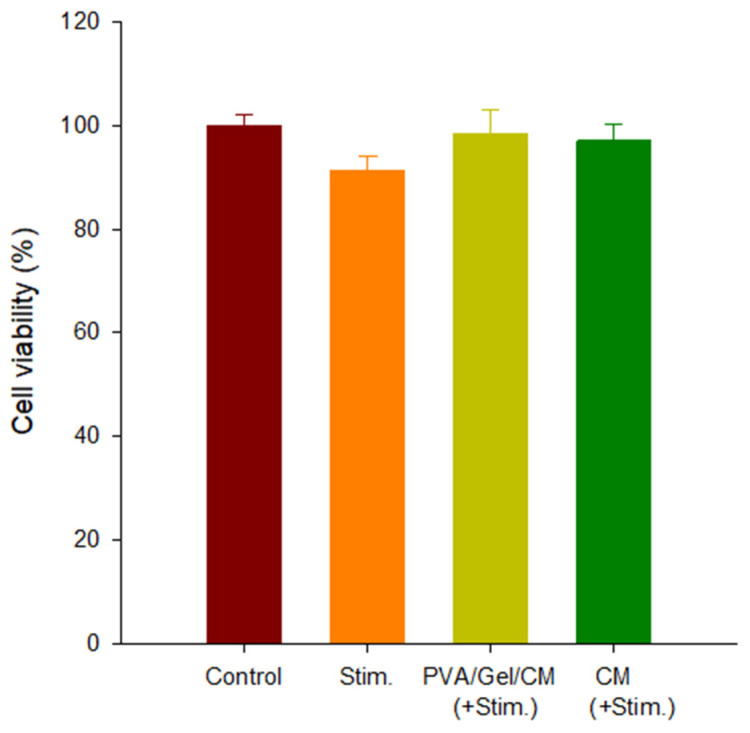
Cell viability of HaCaT cells under different conditions. “Stim.” indicates cells stimulated with inflammatory agents, “PVA/Gel/CM (+Stim.)” indicates cell treated with the PVA/Gel/CM scaffold and stimulants and “CM (+Stim.)” indicates cells treated with CM extract alone and stimulants. Data are presented as mean ± standard deviation. No statistically significant differences were observed between groups (ANOVA, Duncan’s multiple range test).

Figure 5 presents the MTT assay results assessing the cytotoxicity of the PVA/Gel/CM composite hydrogel in HaCaT cells. The following four experimental groups were analyzed: the control group (PVA/Gel); the group stimulated with inflammatory agents IFN-γ and TNF-α (Stim.); the group treated with the PVA/Gel/CM scaffold under inflammatory conditions (PVA/Gel/CM + Stim.); and the group treated with CM alone under inflammatory conditions (CM + Stim.).

The control group (PVA/Gel) exhibited the highest cell viability, reflecting the inherent biocompatibility of the PVA/Gel scaffold. The stimulated group demonstrated a slight decrease in cell viability compared to the control, confirming the cytotoxic effects of the IFN-γ and TNF-α treatment on HaCaT cells [23,24]. However, the addition of the PVA/Gel/CM composite hydrogel (PVA/Gel/CM + Stim.) and CM (+Stim.) exhibited improved cell viability compared to the stimulated group, suggesting that both treatments did not induce cytotoxic effects. This enhanced cell viability is attributed to the bioactive compounds released from CM incorporated into the scaffold. Osthole and imperatorin, key bioactives in CM, are known to exhibit anti-inflammatory and protective properties, mitigating the cellular damage caused by inflammatory agents. By facilitating cell recovery and promoting proliferation, the PVA/Gel/CM scaffold demonstrates its potential as a therapeutic material for inflammatory skin disorders such as atopic dermatitis.

These findings underscore the PVA/Gel/CM composite hydrogel’s biocompatibility and its ability to counteract inflammation-induced cytotoxicity, validating its role as a multifunctional scaffold for tissue engineering applications.

### 2.6. Result of DAPI Stain

**Figure 6:**
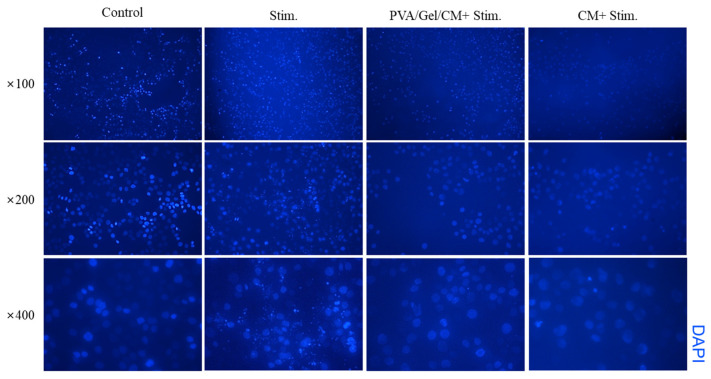
DAPI staining in HaCaT cells under different conditions at magnifications of ×100, ×200, and ×400. “Stim.” indicates cells stimulated with inflammatory agents, “PVA/Gel/CM (+Stim.)” indicates cell treated with the PVA/Gel/CM scaffold and stimulants and “CM (+Stim.)” indicates cells were treated with the CM extract alone and stimulants.

Figure 6 displays the results of DAPI staining, used to qualitatively assess the biocompatibility and cytotoxicity of the PVA/Gel/CM composite scaffold in HaCaT cells [25]. The staining results highlight differences in nuclear morphology and cell density across the following four experimental conditions: the control group; the group stimulated with inflammatory agents IFN-γ and TNF-α (Stim.); the group treated with the PVA/Gel/CM scaffold under inflammatory conditions (PVA/Gel/CM + Stim.); and the group treated with CM alone under inflammatory conditions (CM + Stim.).

The control group, which was not exposed to inflammatory stimuli, exhibited a high density of evenly distributed nuclei, indicating a healthy and confluent cell layer [26]. In contrast, the Stim. group showed a marked reduction in cell density, with sparsely distributed nuclei, reflecting the cytotoxic effects of IFN-γ and TNF-α on HaCaT cells [27]. Notably, cells treated with the PVA/Gel/CM scaffold and CM extract under inflammatory conditions exhibited increased cell density and nuclear clustering, suggesting that the scaffold provided a protective microenvironment that promoted cell survival and proliferation despite the inflammatory environment.

This improvement in cell viability and proliferation is likely attributed to the bioactive compounds released from CM within the scaffold. Osthole and imperatorin, known for their anti-inflammatory and antioxidant properties, may have mitigated the cytotoxic effects of the inflammatory agents, supporting cellular recovery and tissue repair. These findings align with the MTT assay results, further validating the biocompatibility and therapeutic potential of the PVA/Gel/CM composite scaffold. Furthermore, the similarity between the DAPI images of cells cultured on the fabricated CM-incorporated crosslinked scaffold and those treated directly with CM extract suggests that CM was released from the scaffold over time, exerting a positive effect on cell proliferation and viability in the inflammatory environment. This enhanced cellular response underscores the scaffold’s potential as a regenerative platform for inflammatory skin conditions, particularly atopic dermatitis, where biocompatibility and anti-inflammatory properties are crucial for effective treatment.

### 2.7. Anti-Inflammatory Effects of PVA/Gel/CM on IL-8 Expression

**Figure 7:**
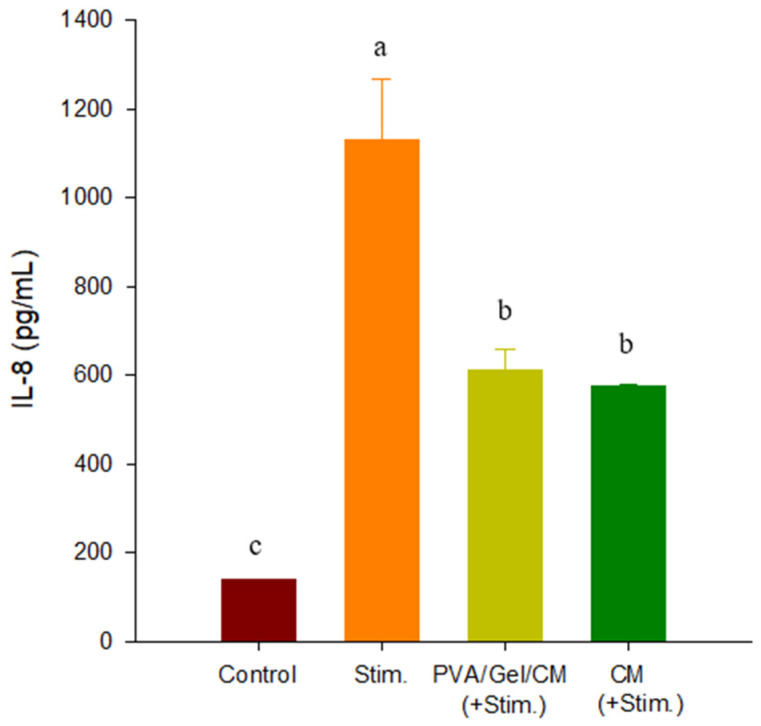
IL-8 production in HaCaT cells under different conditions. “Stim.” indicates cells stimulated with inflammatory agents, “PVA/Gel/CM (+Stim.)” indicates cell treated with the PVA/Gel/CM scaffold and stimulants and “CM (+Stim.)” indicates cells were treated with CM extract alone and stimulants. Data are presented as mean ± standard deviation. Different letters indicate significant differences (*p* < 0.05) by one-way ANOVA followed by Duncan’s multiple range test.

Figure 7 shows the levels of interleukin-8 (IL-8) expression in HaCaT cells under four conditions: the control group; the group stimulated with inflammatory agents IFN-γ and TNF-α (Stim.); the group treated with the PVA/Gel/CM scaffold under inflammatory conditions (PVA/Gel/CM + Stim.); and the group treated with CM alone under inflammatory conditions (CM + Stim.).

The control group showed the lowest IL-8 levels, consistent with the absence of inflammatory stimulation [22]. The Stim. group exhibited a significant increase in IL-8 expression (*p* < 0.05), confirming the induction of inflammatory responses by IFN-γ and TNF-α [23]. However, cells cultured with the PVA/Gel/CM scaffold and CM extract under inflammatory conditions showed a 46% and 49% reduction in IL-8 expression, respectively, compared to the Stim. group, indicating a potent anti-inflammatory effect of the scaffold [24].

The reduction in IL-8 expression in the PVA/Gel/CM + Stim. Group suggests that CM contains bioactive compounds that effectively modulate inflammatory signaling pathways [18,19,20,21]. By mitigating IL-8 production, these compounds likely interfere with cytokine-medicated immune activation, thereby reducing neutrophil recruitment and dampening the inflammatory cascade [22]. The controlled release of CM bioactives from the scaffold ensures a sustained therapeutic effect, making it particularly effective in inflammatory skin conditions like atopic dermatitis (AD). These findings demonstrate the dual functionality of the PVA/Gel/CM scaffold, combining anti-inflammatory properties with biocompatibility and structural integrity. The significant reduction in IL-8 expression highlights its potential as a therapeutic platform for AD and other inflammatory conditions, where the suppression of pro-inflammatory cytokines is essential for effective treatment. Further investigations into the release kinetics of CM bioactives and their long-term effects on inflammatory pathways could provide deeper insights into the scaffold’s therapeutic efficacy.

## 3. Conclusions

This study successfully developed a novel PVA/Gel/CM composite hydrogel scaffold to address the dual challenges of tissue regeneration and inflammation management in atopic dermatitis (AD). Incorporating gelatin into the PVA matrix enhanced cell adhesion and proliferation, while bioactive compounds from CM, such as osthole and imperatorin, significantly improved gelation efficiency and provided potent anti-inflammatory effects. The scaffold’s porous structure, achieved through optimized freeze–thaw crosslinking and lyophilization, facilitated the controlled release of bioactive compounds, as confirmed by UPLC analysis.

Comprehensive physicochemical and biological assessments demonstrated the scaffold’s superior properties. FT-IR analysis revealed critical structural modifications driven by interactions among the PVA, gelatin, and CM; SEM imaging highlighted a highly developed porous network conducive to cell growth and drug delivery. Biological evaluations using HaCaT cells under IFN-γ and TNF-α-induced inflammatory conditions showed that the PVA/Gel/CM composite hydrogel significantly improved cell viability and proliferation. Furthermore, the scaffold effectively reduced IL-8 expression in stimulated HaCaT cells, underscoring its strong anti-inflammatory potential.

These findings position the PVA/Gel/CM composite hydrogel as a promising candidate for treating inflammatory skin conditions like AD. Its combination of biocompatibility, structural integrity, and sustained therapeutic release establishes it as an advanced platform for tissue engineering and regenerative medicine applications.

Future research will focus on elucidating the molecular mechanisms underlying the scaffold’s anti-inflammatory effects and evaluating its efficacy in in vivo models. Additionally, expanding its application to broader inflammatory and degenerative diseases will further validate its clinical potential. This work provides a foundation for developing next-generation biomaterials capable of combining regenerative and therapeutic functionalities, addressing critical gaps in the treatment of complex inflammatory disorders.

## 4. Materials and Methods

### 4.1. Materials and Chemicals

The PVA used in the experiments was grade F-17 from OCI Inc., Republic of Korea, with a polymerization degree of 1700, a crystallinity of 98–99.5%, and a molecular weight of 74,800 g/mol. The gelatin used was Type B gelatin derived from bovine skin, purchased from Sigma–Aldrich (G-6650, St. Louis, MO, USA). Additionally, dried fruits of CM were extracted with methanol at a ratio of 20:1 (*w*/*v*) for 24 h at room temperature; this process was repeated twice. The resulting extract was concentrated and converted into a powder form, which was purchased from Human Hurb Co. (Daegu, Republic of Korea) and used for the experiments.

### 4.2. Preparation of PVA/Gelatin/CM Hydrogel Scaffold

To fabricate PVA/Gelatin/CM (PVA/Gel/CM) composite scaffolds for the regeneration of skin tissue affected by atopic dermatitis, a hydrogel incorporating bioactive natural compounds was developed. Polyvinyl alcohol (PVA), a biocompatible and biodegradable polymer, served as the primary material, while gelatin (Gel) was incorporated to improve cell adhesion properties. Furthermore, CM, renowned for its anti-inflammatory characteristics, was processed via methanol extraction to obtain its total extract. The resulting PVA/Gel/CM composite hydrogel was specifically designed to function as a drug-releasing scaffold, leveraging the bioactive compounds derived from natural sources to promote therapeutic effects.

**Figure 8:**
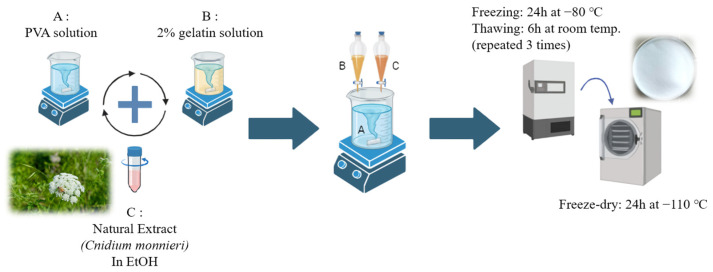
Schematic diagram of PVA/Gel/CM hydrogel scaffold manufacturing method.

The fabrication process of the PVA/Gel/CM composite hydrogel dressing patches and scaffolds is illustrated in Figure 8. A 10% aqueous solution of PVA was prepared, while gelatin was dissolved in water to create a 2% solution. The CM extract was dissolved in ethanol at a concentration of 20 μL/mL. During the mixing process, droplets of the gelatin and CM extract solutions were added to the PVA solution under continuous stirring using a magnetic stirrer. After 30 min of stabilization, the PVA/Gel/CM hydrogel was formed.

To produce the final porous hydrogel, the prepared hydrogel mixture was poured into 35 mm Petri dishes to mold its shape. The samples underwent three cycles of freeze-thawing, where they were frozen at −80 °C for 24 h and then thawed at room temperature for 6 h. This process facilitated physical crosslinking between the molecular chains. Finally, the hydrogels were freeze-dried to create a porous structure containing the natural compounds.

The samples were prepared as follows: the control sample (PVA/Gel) was fabricated by mixing only the PVA solution with the gelatin solution. The stimulated sample was derived from the control sample for subsequent analysis. Furthermore, a PVA/Gel/CM sample was prepared by adding 40 μg/mL of CM extract to the PVA/Gel control sample. Comparative studies were conducted to evaluate the properties and performance of these samples.

### 4.3. FT-IR and Compreesive Strength Analysis

To analyze the structural changes in the prepared hydrogels, an FTIR spectrometer (Nicolet iS20, Thermo Fisher Scientific, Waltham, MA, USA) was used. The spectra were measured over a wavenumber range of 4000–600 cm^−1^ with a resolution of 4 cm^−1^.

The compressive strength was measured using an AGS-X universal testing machine (Shimadzu Co., Kyoto, Japan) at room temperature. During the test, the crosshead speed was set to 10 mm/min, and values were measured when the specimens reached 50% deformation. The measurements were repeated three times and the average value was used for analysis.

### 4.4. Gel Content and Swelling Ratio

To assess the gel content and swelling ratio of the hydrogel samples, a sequential washing, drying, and weight measurement process was conducted. For gel content determination, unreacted polymers that did not participate in the crosslinking reaction were removed by continuously stirring and washing the hydrogel samples at room temperature for 24 h. Following the washing process, the hydrogels were carefully removed, excess surface moisture was blotted away, and the samples were dried in an oven at 4 °C for 24 h. The gel content (%) was then calculated as the ratio of the dried hydrogel weight (W_d_) to the initial polymer weight (W_i_), as described in Equation (1), as follows:

Gel content (%) = W_d_/W_i_ × 100 (1)

Each measurement was performed five times, and the average value was used for analysis. For the swelling ratio evaluation, the hydrogel was first dried at 40 °C for 24 h and its initial dry weight (W_a_) was recorded. The dried sample was then immersed in distilled water at room temperature for 48 h, after which its swollen weight (W_s_) was measured. Each measurement repeated five times and the average value was used. The swelling ratio (%) was determined using Equation (2), as follows:

Swelling ratio (%) = W_s_/W_a_ × 100 (2)

### 4.5. SEM Analysis

The morphology of the PVA/Gel/CM hydrogel scaffolds, including cross-sectional and side-sectional structures, was examined using a TM4000PlusII scanning electron microscope (Hitachi, Tokyo, Japan).

### 4.6. UPLC Analysis

The UPLC analysis was performed using an Agilent 1290 Infinity II system (Agilent Technologies, Santa Clara, CA, USA), equipped with a Phenomenex Kinetex 1.7 µm XB-C18 column (100 Å, 50 × 2.1 mm) to achieve chromatographic separation. To protect the analytical column and ensure optimal performance, a SecurityGuard ULTRA guard column (UHPLC C18 for 2.1 mm, Phenomenex, Torrance, CA, USA) was utilized. The column temperature was consistently maintained at 40 °C to ensure reliable and reproducible chromatographic behavior. The mobile phase consisted of two solvents: Solvent A, acetonitrile, and Solvent B, water containing 0.1% formic acid (Daejung Chemicals & Metals co., Gyeonggi-do, Republic of Korea). A gradient elution method was employed to facilitate the efficient separation of analytes. The flow rate was set at 0.3 mL/min to optimize the peak resolution and ensure stable retention times. An injection volume of 1 µL was used for each sample, prepared at a concentration of 0.02 mg/mL. The gradient elution began with 30% of Solvent A and 70% of Solvent B, held constant for 0.3 min. Between 0.3 and 8 min, the proportion of Solvent A was gradually increased to 80%, with a corresponding decrease in Solvent B. At 8.5 min, Solvent A was increased to 100%, completely replacing Solvent B. The system was maintained at 100% Solvent A until the end of the run at 14 min, ensuring the complete elution of all analytes from the column. The detection wavelength was set at 300 nm for analysis.

### 4.7. Cumulative Release

The release profile of CM from the PVA/Gel/CM hydrogel was evaluated in a DMEM medium at 37 °C. To monitor the release kinetics, approximately 50% of the total solution volume was periodically withdrawn from the release medium. To maintain a constant total volume throughout the experiment, a fresh DMEM medium was replenished following each sampling. A calibration curve was established using UPLC analysis at 300 nm, with CM total extract concentrations of 1, 0.5, 0.25, 0.125, and 0.0625 mg/mL. The quantification was conducted based on the absorbance of osthole, the primary bioactive compound present in the CM extract. The cumulative release amount of CM was determined based on UV spectrophotometric measurements at 300 nm, using the following equation [28]:

(3) $$\mathrm{Cumulative}\;\mathrm{release}\;(\%)=C_{n}V+\sum_{i=1}^{i=n-1}C_{i}V_{i}$$

where *V* represents the total volume of the release solution and *V_i_* denotes the volume of the sampled solution. *C_n_* and *C_i_* indicate the concentration of osthole in the CM within the release solution and the sampled solution, respectively. The experiment was repeated three times and the results were expressed as mean and standard deviation.

### 4.8. Cell Culture and Cell Cytotoxicity

HaCaT cells, obtained from Cell Lines Service (Eppelheim, Germany), were cultured in Dulbecco’s Modified Eagle Medium (DMEM, GenDEPOT, Barker, TX, USA), supplemented with 10% fetal bovine serum (FBS; Gibco, ON, Canada) and 1% penicillin–streptomycin (Gibco BRL, Grand Island, NY, USA). To evaluate the viability of HaCaT cells cultured on hydrogel scaffolds, the cells were maintained in the same medium and incubated at 37 °C in a 5% CO_2_ humidified incubator. Cell viability was evaluated through the MTT assay. HaCaT cells were plated in 96-well plates containing the scaffolds at a density of 5 × 10⁴ cells/well and cultured for 24 h. To stimulate an inflammatory response, 5 ng/mL of IFN-γ (PeproTech, Rocky Hill, NJ, USA) and 5 ng/mL of TNF-α (PeproTech) were added to the culture. After an additional 24 h of incubation, 10 μL of MTT solution (5 mg/mL, Sigma–Aldrich) was added into each well, and the plate were incubated at 37 °C for 4 h. Subsequently, the medium was removed, and 100 μL of DMSO (Sigma–Aldrich) was added and the plate was left to incubate at room temperature for 20 min; the absorbance was measured at 450 nm using a microplate reader (AMR-100, Allsheng, Hangzhou, China).

### 4.9. DAPI Stain

DAPI staining was performed to observe morphological changes in cell nuclei. The procedure was identical to the 4.8 MTT assay for the first two days. On the third day, the cells were washed twice with PBS and fixed with 4% formaldehyde (Biosesang, Seoul, Republic of Korea) for 15 min. After fixation, the cells were washed once more with PBS. DAPI solution (Thermo Fisher Scientific) was then added and the cells were incubated at room temperature for 30 min. Following incubation, the cells were washed with PBS and observed under a fluorescence microscope (Eclipse Ts2R, Nikon Inc., Tokyo, Japan).

### 4.10. Interleukin-8 Enzyme-Linked Immunosorbent Assay

HaCaT cells were cultured under the same conditions as described in the previous protocol. Afterwards, the cell culture supernatants were collected and stored at −80 °C for subsequent analysis. The concentration of IL-8 was measured using an enzyme-linked immunosorbent assay (ELISA) kit (R&D Systems, Minneapolis, MN, USA), following the guidelines provided by the manufacturer.

### 4.11. Statistical Analysis

The experimental data were analyzed using SAS software (version 9.4; SAS Institute, Cary, NC, USA). A one-way analysis of variance (ANOVA) was performed, followed by Duncan’s multiple range test to determine statistical significance.

## Data Availability

The original contributions presented in this study are included in the article. Further inquiries can be directed to the corresponding author.
